# Supplementary material for: Willingness to vaccinate against COVID-19 among Bangladeshi adults: Understanding the strategies to optimize vaccination coverage
Source: PLoS One. 2021 Apr 27;16(4):e0250495. doi: 10.1371/journal.pone.0250495 (PMC8078802; doi:10.1371/journal.pone.0250495)
Supplement: S1 File — (DOCX) [file pone.0250495.s001.docx]

Table of Contents

[S1 Table: Sample allocation across 8 districts of Bangladesh 2](#_Toc67647454)

[S1 Appendix: Calculation of Response rate 2](#_Toc67647455)

[S2 Table: Logistic model with vaccine hesitancy 4](#_Toc67647456)

[S3 Table: Logistic model with vaccine unwillingness 5](#_Toc67647457)

[S1 Fig. Vaccine acceptance with cost by occupational groups 6](#_Toc67647458)

[S2 Fig. Vaccine acceptance with cost by location of residence 7](#_Toc67647459)

# S1 Table: Sample allocation across 8 districts of Bangladesh

|  | \| Region \| District \| Target participants \| Respondents \| \| --- \| --- \| --- \| --- \| \| City \| Dhaka \| 800 \| 626 \| \| City \| Chattogram \| 800 \| 641 \| \| Rural \| Jamalpur \| 500 \| 388 \| \| Upazila and Rural \| Rangpur \| 500 \| 413 \| \| Upazila and Rural \| Dinajpur \| 500 \| 406 \| \| Upazila and Rural \| Rajshahi \| 500 \| 391 \| \| Upazila and Rural \| Khulna \| 500 \| 383 \| \| Rural \| Pirojpur \| 500 \| 398 \|   Response rate 79.3%. |
| --- | --- | --- | --- | --- | --- | --- | --- | --- | --- | --- | --- | --- | --- | --- | --- | --- | --- | --- | --- | --- | --- | --- | --- | --- | --- | --- | --- | --- | --- | --- | --- | --- | --- | --- | --- | --- | --- |

# S1 Appendix: Calculation of Response rate

The response rate (RR) is the number of complete interviews with reporting units divided by the number of eligible reporting units in the sample. The following formula was used to calculate the response rate [1].


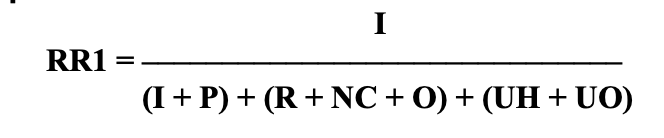


Where, RR1 = Response rate

I = Complete interview

P = Partial interview

R = Refusal and break-off

NC = Non-contact

O = Other

UH = Unknown if household/occupied HU

UO = Unknown, other

The details of these quantities for our study are given in the following:

I and P=The in-person household survey was conducted in which housing units are sampled from an address-based sampling frame of eight selected camps using systematic sampling technique. We consider less than 50% of all applicable questions answered (with other than a refusal or no answer) equals break-off, 50%-80% equals partial, and more that 80% equals complete. We found complete answered from 3646 participants (i.e., I=3646). We found 78 of the participants did not complete the questionnaire for PTSS (i.e., P=78).

R= Refusals and break-offs consist of cases in which some contact has been made with the housing unit and a responsible household member has declined to do the interview, or an initiated interview results in a terminal break-off. Six hundred and thirty-seven of the respondents refused to take part in the interview, but no one took a terminal break-off after initiating an interview (i.e., R=637).

NC= Non-contacts in in-person household surveys consist of three types: a) unable to gain access to the building, b) no one reached at housing unit, and c) respondent away or unavailable. 76 housings were not accessible in few districts due to security condition, and we were not able to reach at housing unit during the interview period (i.e., NC=76).

O= Other cases represent instances in which the respondent is/was eligible and did not refuse the interview, but no interview is obtainable because of: a) death; b) the respondent is physically and/or mentally unable to do an interview; c) language problems; and d) miscellaneous other reasons. We did not face any language problem to exclude participants. We found 21 physically ill (bed-bound or palliative care) patients from household during the interview (i.e., O=21).

UH= Cases of unknown eligibility and no interview include situations in which it is not known if an eligible housing unit exists and those in which a housing unit exists, but it is unknown whether an eligible respondent is present in the housing unit. It was not a scenario in our situation (i.e., UH=0).

UO= Not eligible cases for in-person household surveys include: a) out-of-sample housing units; b) not-a-housing unit; c) vacant housing units; d) housing units with no eligible respondent; and e) situations in which quotas have been filled. In a systematic sampling technique, we found a total of 142 households without any adult respondents during the interview (UO=142).

Thus, the response rate is, RR=3646/ (3646+78+637+76+21+0+142) = 79.3%

**Reference:**

[1] The American Association for Public Opinion Research. 2016. *Standard Definitions: Final Dispositions of Case Codes and Outcome Rates for Surveys. 9th edition*. AAPOR.

# S2 Table: Logistic model with vaccine hesitancy


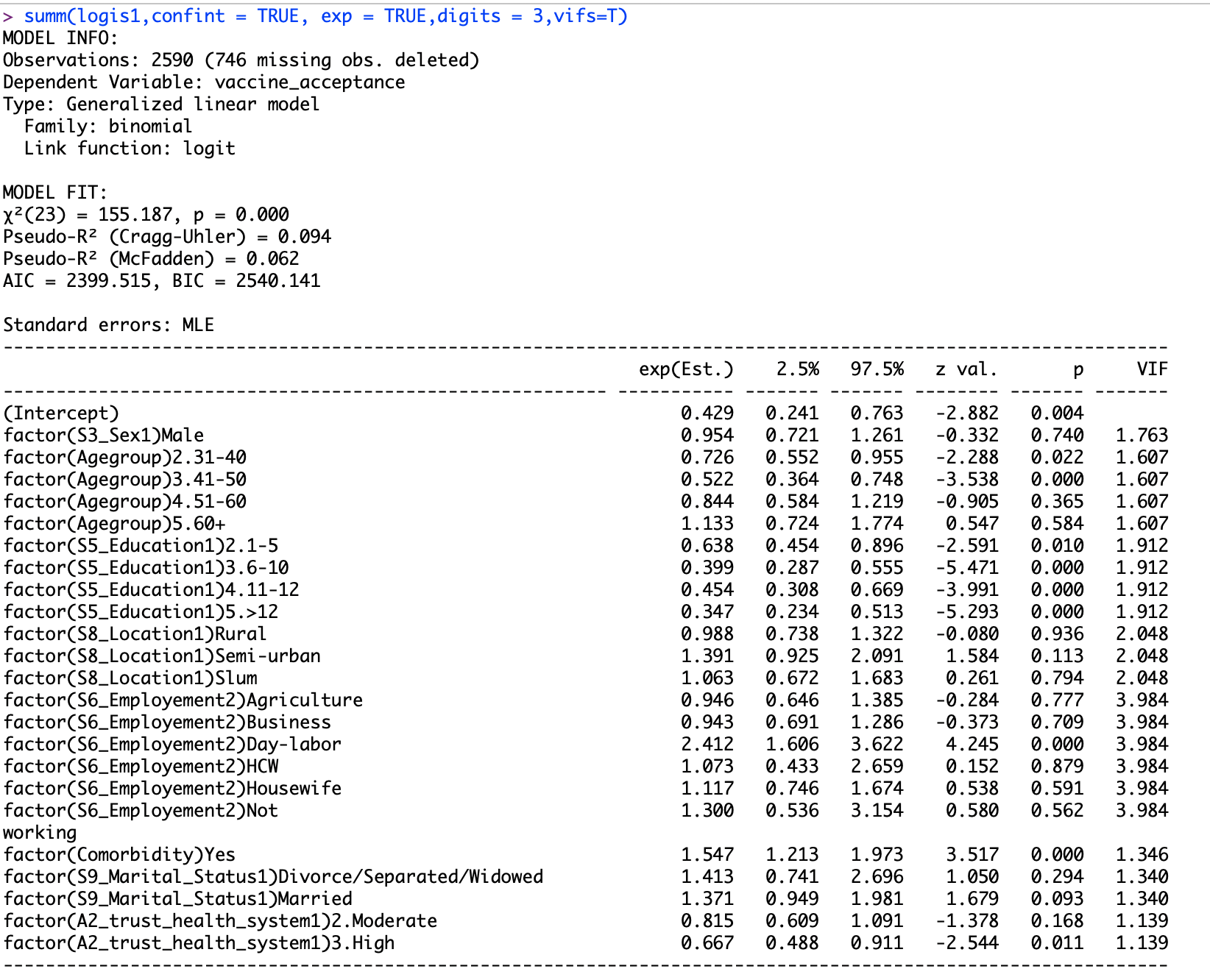


# S3 Table: Logistic model with vaccine unwillingness


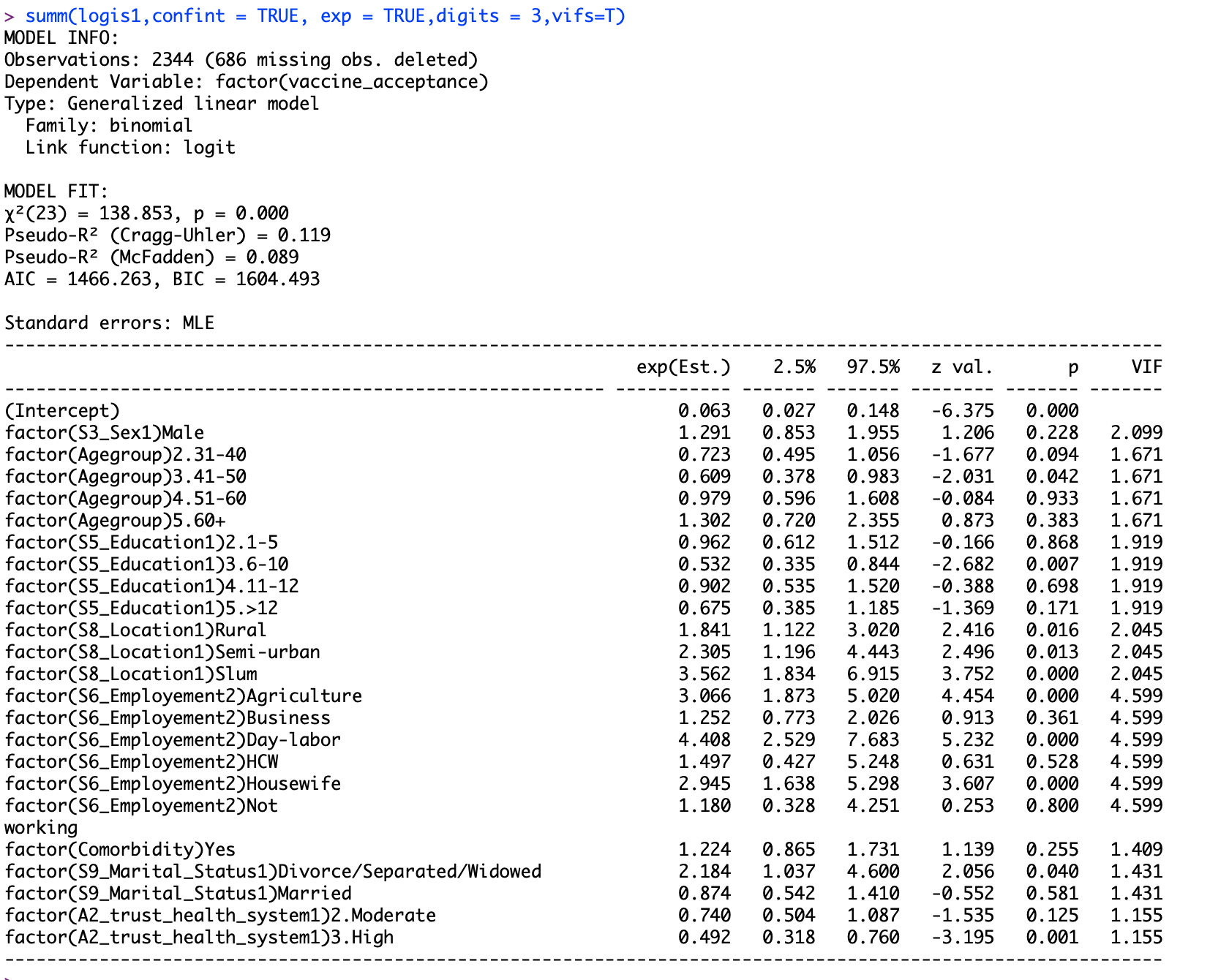


# S1 Fig. Vaccine acceptance with cost by occupational groups

# S2 Fig. Vaccine acceptance with cost by location of residence
