# Supplementary material for: Willingness to vaccinate against COVID-19 among Bangladeshi adults: Understanding the strategies to optimize vaccination coverage
Source: PLoS One. 2021 Apr 27;16(4):e0250495. doi: 10.1371/journal.pone.0250495 (PMC8078802; doi:10.1371/journal.pone.0250495)
Supplement: Attachment — Submitted filename: PLOS Review report.docx [file pone.0250495.s002.docx]

Review report

This study assesses National trends in the willingness to vaccinate against COVID-19 among Bangladeshi adults: Understanding the strategies to optimize vaccination coverage. This is an interesting study and indicated the common public problems of the residents in Bangeladish.

Minor comments

- You have assessed the proportion of residents who are willing to vaccinate against COVID-19, not a trend. Therefore; better to revise your title accordingly.
- As a researcher, the term “methodology” is a broad science, you did not address with this single research. Therefore, replace with the term “methods and materials”.
- In methodology, the first paragraph is too long. Therefore, either minimize it to 6-8 lines or split it into certain paragraphs.
- Please feed the source and study populations of this study.
- How did you manage more than one household heads found in one compound (such as rent)?
- Is there any plan of revisit for the households closed during data collection?
- Is it possible to calculate wealth index (PCA) rather than monthly income?
